# T171. REDUCED FRONTAL CORTICAL THICKNESS AND SURFACE IN A 10 YEARS FOLLOW-UP OF EARLY ONSET PSYCHOSIS

**DOI:** 10.1093/schbul/sby016.447

**Published:** 2018-04-01

**Authors:** Daniel Ilzarbe, Elena de la Serna, Inmaculada Baeza, Jose Pariente, Adriana Fortea, Marina Redondo, Nuria Bargallo, Josefina Castro-Fornieles, Gisela Sugranyes

**Affiliations:** 1 Hospital Clinic de Barcelona; 2 Hospital Clinic of Barcelona, Centro de Investigación Biomédica en Red de Salud Mental (CIBERSAM); 3 Hospital Clinic of Barcelona, Centro de Investigación Biomédica en Red de Salud Mental (CIBERSAM), Institut d’Investigacions Biomèdiques Agustí Pi i Sunyer (IDIBAPS); 4 Magnetic Resonance Image Core Facility, Institut d’Investigacions Biomèdiques Agustí Pi i Sunyer (IDIBAPS); 7 Hospital Clinic of Barcelona, Institut d’Investigacions Biomèdiques Agustí Pi i Sunyer (IDIBAPS)

## Abstract

**Background:**

Structural volume loss of cortical gray matter over time in schizophrenia has been widely reported (Vita et al. 2012), and may be more pronounced when the disorder has an onset prior to age 18 (Early Onset Psychosis, EOP; Arango et al. 2008). More recently, studies have focused on measures of cortical morphology. The single study in EOP so far has identified greater loss of cortical thickness (CTH) in patients with schizophrenia over time (van Haren et al. 2011), whereas to our knowledge, no so far study has examined measures of surface area (SA) in EOP following a longitudinal design. We set out to examine measures of both CTH and SA in a sample of EOP at 10-year-follow-up.

**Methods:**

Patients with EOP were recruited at first episode, matched by sex and age with healthy controls (HC) and re-assessed at 10 years. Subjects were evaluated clinically and structural T1 volumes were acquired using magnetic resonance imaging at baseline and 10-year-follow-up. Images were preprocessed, segmented and analysed with FreeSurfer. Quality control procedure was carried out by two raters. Images were segmented and CTH and SA values were extracted for each parcellation employing Desikan-Killiany Atlas; these were grouped in frontal, occipital, temporal, parietal and cingulate lobes so as to reduce multiple comparisons. When group or group by time effects were detected, parcellations were individually examined. A linear mixed model was built using Stata IC 13.1 to evaluate the effect of group and time on CTH and SA, including hemisphere as fixed effects and correcting by total intracranial volume and setting a critical p-value of .05.

**Results:**

Thirty-nine subjects completed the follow-up. After removing 9 due to poor quality T1 images (technical problems, excess of movement), 28 subjects were finally included (13 EOP, 15 HC). There were no significant differences in age (EOP=26.9 ± 0.6 vs HC=27.2 ± 0.3 at follow-up) or sex distribution (%female: EOP=43% vs HC=38%) between groups. The distribution of diagnosis in the case group was: schizoaffective disorder (n=5), bipolar disorder with psychotic features (n=3), schizophrenia (n=2) and others (n=3).

There was a trend-level group effect in global CTH (p = .07) which was significant in the frontal lobe (p = .014). EOP exhibited less CTH in the caudal middle frontal (p = .016) and pars opercularis (p = .03) and orbitalis (p = .007) of the inferior frontal gyrus. There was an effect of time in the parietal (p = .013) and occipital (p = .004) lobes consisting of thinner CTH at follow-up in both groups.

There were no differences in SA between groups. Both showed an increase in total SA (p < .001) and for parietal (p < .001), temporal (p = .009) and occipital (p = .003) regions at follow-up. There a group by time effect in frontal SA, consisting of an increase over time in HC and a decrease in EOP (p = .044), specifically in medial orbito-frontal cortex (p = .039).

**Discussion:**

Our results have identified: 1) thinner cortices in frontal regions in EOP compared to HC, which seems to be constant over time; and 2) a decreased in SA in frontal areas in EOP along time, contrasting with HC, whose frontal surface increased at follow-up. These findings are consistent with another study (Greenstein et al. 2006) which also reported reduced CTH in frontal areas in EOP during development, while we found no abnormalities in temporal regions (Vita et al. 2012). Despite the small sample size, to our knowledge this is the longest follow-up of an EOP sample employing magnetic resonance imaging so far.

